# From papers to RDF-based integration of physicochemical data and adverse outcome pathways for nanomaterials

**DOI:** 10.1186/s13321-024-00833-0

**Published:** 2024-05-01

**Authors:** Jeaphianne P. M. van Rijn, Marvin Martens, Ammar Ammar, Mihaela Roxana Cimpan, Valerie Fessard, Peter Hoet, Nina Jeliazkova, Sivakumar Murugadoss, Ivana Vinković Vrček, Egon L. Willighagen

**Affiliations:** 1 Dept of Bioinformatics, BiGCaT, NUTRIM, FHML, Maastricht University, Maastricht, The Netherlands; 2 Department of Clinical Dentistry, Faculty of Medicine, University of Bergen, Bergen, Norway; 3 Fougères Laboratory, Anses, French Agency for Food, Environmental and Occupational Health and Safety, Toxicology of Contaminants Unit, Fougères, France; 4 Laboratory of Toxicology, Unit of Environment and Health, Department of Public Health and Primary Care, KU Leuven, Leuven, Belgium; 5 Ideaconsult Ltd., Sofia, 1000 Bulgaria; 6 SD Chemical and Physical Health Risks, Brussels, Belgium; 7 Institute for Medical Research and Occupational Health, Zagreb, Croatia

**Keywords:** Nanosafety, Resource description framework, Adverse outcome pathways, Engineered nanomaterials

## Abstract

**Abstract:**

Adverse Outcome Pathways (AOPs) have been proposed to facilitate mechanistic understanding of interactions of chemicals/materials with biological systems. Each AOP starts with a molecular initiating event (MIE) and possibly ends with adverse outcome(s) (AOs) via a series of key events (KEs). So far, the interaction of engineered nanomaterials (ENMs) with biomolecules, biomembranes, cells, and biological structures, in general, is not yet fully elucidated. There is also a huge lack of information on which AOPs are ENMs-relevant or -specific, despite numerous published data on toxicological endpoints they trigger, such as oxidative stress and inflammation. We propose to integrate related data and knowledge recently collected. Our approach combines the annotation of nanomaterials and their MIEs with ontology annotation to demonstrate how we can then query AOPs and biological pathway information for these materials. We conclude that a FAIR (Findable, Accessible, Interoperable, Reusable) representation of the ENM-MIE knowledge simplifies integration with other knowledge.

**Scientific contribution:**

This study introduces a new database linking nanomaterial stressors to the first known MIE or KE. Second, it presents a reproducible workflow to analyze and summarize this knowledge. Third, this work extends the use of semantic web technologies to the field of nanoinformatics and nanosafety.

**Supplementary Information:**

The online version contains supplementary material available at 10.1186/s13321-024-00833-0.

## Introduction

With engineered nanomaterials (ENMs) playing an increasingly important role in our lives, the need for adequate risk assessments (RA) for ENMs is urgent [1–3]. The broad diversity of ENMs and the wide range of their applications, from paints or coatings to medicine, food and cosmetics, introduce both opportunities and challenges. New applications and nano-enabled products are regularly introduced on the market, solving problems such as antimicrobial resistance [4–7], safer drug formulations and delivery [8–10] and cost-efficient sensor technologies [11, 12], to name a few. However, the diversity in ENM design and applications has been raising many concerns related to hazard and exposure assessment whenever detailed quality and safety data are lacking. The concern is even bigger due to a lack of integration of cumulative hazards and aggregate exposure assessment in the current RA framework that underpins existing regulation. Moreover, differences in legislated procedures for different ENMs classes (e.g., pharma-grade, industrial, advanced materials) [13] further complicate matters.

Currently, the main methodologies for RA of chemicals/materials mostly rely on in vivo (animal) testing. The limited in vivo toxicity data, coupled with the recent paradigm shift in toxicity testing towards the development and use of pathway-based testing strategies rather than traditional animal–based methods, triggered interest in the development of New Approach Methodologies (NAMs). These methodologies are based on *in chemico*, in vitro, in silico*,* and alternative species test methods. This reinforces the implementation of the 3R’s (Replacement, Reduction and Refinement of animals) concept [14]. Although NAMs are not envisioned as a direct replacement for in vivo testing, it offers an opportunity for a fit-for-purpose testing strategy in the context of an Integrated Approach to Testing and Assessment (IATA) framework to inform decision-making bodies. It is envisioned that the NAMs should strategically be accompanied by the Adverse Outcome Pathway (AOP) concept [15, 16], which has been formalized by the OECD in 2012 as the basis for organizing data and developing an IATA [17, 18].

An AOP starts with a molecular initiating event (MIE), usually followed by a series of sequential and causal key events (KEs) at cellular, tissue or organ levels of biological organisation and ends with adverse outcome(s) (AOs) defined as the disease or the pathological outcomes, which are targeted for regulatory decisions. These pathways allow systematic collection, organization, and simpler presentation of the information collected from in vitro, in vivo or in silico toxicological studies. As AOPs are not chemical-specific, they can be used in the identification of key toxicological data gaps and prioritization of research efforts and resources, thus supporting IATA [19]. When biological interaction and exposure for fully characterized ENM-relevant stressors are identified, existing AOPs may thus facilitate regulatory-relevant RA.

On a handful of toxicological endpoints triggered by ENMs, such as oxidative stress and inflammation, there is an abundance of published data. However, for the many other endpoints, information on the identification of which AOPs are relevant or specific to ENMs, and understanding the ENM-related molecular processes underlying the KEs is sparse or missing. This might be due to the lack of clear and centralized data and knowledge on the identification of ENMs, their characteristics and their interaction with biological systems. The management of these (toxicological) data needs to operate under FAIR (findability, accessibility, interoperability, and reusability) principles for effective RA and hazard assessment [20–22].

One way to achieve this would be to make the data more machine-readable. The use of Semantic Web Technologies (SWTs), such as the Web Ontology Language and Resource Description Framework (RDF), is a possible method. RDF uses ontologies to describe data, define properties and introduce relations between them (for example the characteristics of an ENM). The standardized vocabulary introduced by ontologies enables the communication between databases, in line with the FAIR principles, and makes the information machine-readable. RDF enriched with ontologies can be used to combine information from different resources and answer complex biological questions.

RDF is applied in various resources for the storage of data and information relevant for RA and hazard assessment. For example, the AOP-Wiki RDF stores mechanistic toxicological knowledge and provides additional molecular descriptors. Similarly, WikiPathways contains molecular pathways and defines all molecular entities involved [23], neXtProt provides protein-centered knowledge [24, 25], while eNanoMapper enables the storage, searching and sharing of toxicological data for ENMs [26].

Developing RDF for ENMs and their molecular and biological interactions as described in literature, would allow the exploration of available scientific and professional databases for potential risks of ENM exposure. For example, the AOP-Wiki can extrapolate downstream adverse effects from molecular interactions captured in MIE(s) and KE(s). Furthermore, by implementing a standard format, it will be possible to perform correlation analyses on physicochemical properties and biological effects of ENMs. The use of SWTs, including RDF, ontologies, and linked data, plays a pivotal role in addressing communication gaps, achieving semantic interoperability, and facilitating data integration. The SWTs-based approach not only integrates data across different sources and provides an interoperable format for secondary use but also demonstrates the power of ontological modeling in capturing complex relationships within the modeled domain, which would be challenging to achieve through traditional methods. SWTs have been used for a long time in diverse domains such as biomedical informatics and cheminformatics but rarely applied in the field of nanosafety or nanoinformatics.

Here, we present a proof of principle of implementing SWTs for reporting ENMs, their physicochemical characteristics and their interactions at different levels of biological organization. It expands the work initiated by Murugadoss et al. (2021), where a selection of scientific papers was analyzed for ENMs’ connections to AOPs [27]. Twenty-one of the papers investigated in that study were further analyzed to extract detailed information about the 83 unique ENMs, their physicochemical properties, and their potential effects. All materials were registered in the European Registry of Materials (ERM) [28]. The registry allows the creation of a persistent, unique identifier for ENMs to enable correct and precise documentation.

The RDF has been made publicly available through a SPARQL endpoint and a corresponding SNORQL User Interface to facilitate the exploration of the data with SPARQL queries (nanosafety.rdf.bigcat-bioinformatics.org/). This allows us to perform federated SPARQL queries, expanding the developed RDF with information from other sources. Federated SPARQL queries can be used, e.g., to explore the downstream effects of the proposed MIEs in more detail or group nanomaterials based on their ontological annotations.

Here, we present the collected data, the RDF structure and the SPARQL endpoint, demonstrating their applicative nature in answering relevant biological questions.

## Methods

### Literature review and dataset creation

Murugadoss et al. (2021) identified relevant papers using the search terms “adverse outcome pathway’’ OR “AOP” AND “nano^*^,” resulting in 960 papers up to 2020-12-01. After refining for duplicates, reviews, AOPs/AOs reports, and the type of organisms studied, 32 papers covering both in vitro and in vivo studies on mammals were selected, as detailed in Additional file 1: Table S1 [27]. From this collection, our focus narrowed to a subset of 21 papers that include measures of the physicochemical properties of reported ENMs. Each ENM described in the analyzed papers was assigned a new ERM identifier to facilitate information extraction and later enable cross-referencing with other data resources [28]. The RDF Schema specification was used to store information and the ENM types were set to chemical substances using the CHEBI_59999 term from the ChEBI ontology [29]. The new identifier, label, creator information and GitHub page were added to the registry Turtle as in Additional file 1: Fig S1.

The ERM identifiers correspond to the ENMs in the specific study reported in the paper. Some ENMs, e.g., standard materials, can later be mapped to other ERM identifiers that describe exactly the same material. More detailed metadata is available at https://h2020-riskgone.github.io/riskgone-materials/RiskGONE_Literature_NM.html.

To enable grouping within the created dataset, or linking with other datasets, the material types were described by introducing semantic annotations of materials. This was done using the NanoParticle Ontology (NPO) [30, 31], eNanoMapper (eNM) ontology [32] and Chemical Entities of Biological Interest (ChEBI) ontology [29] by exploring BioPortal for the fitting material types, aiming for the most precise descriptions of material types.

The physicochemical properties of each ENM described in these scientific papers were extracted and stored in a collection spreadsheet Additional file 2. These properties include the primary particle size, hydrodynamic diameter, shape, diameter, bundle diameter, surface area and zeta potential amongst others see Additional file 2. Additionally, the characterisation method, and producer and model of the measuring device were reported in the same sheet. The data in the spreadsheet were extended with the reported molecular effects of the ENMs, which were then linked to MIE and KE identifiers from the AOP-Wiki. This association was established through a Jupyter Notebook executing SPARQL queries against the AOP-Wiki RDF and involved manual curation [33].

Finally, the metadata of the papers was manually added to the sheet by accessing the papers using their DOI.

### Creating the nanosafety RDF

**Fig. 1:**
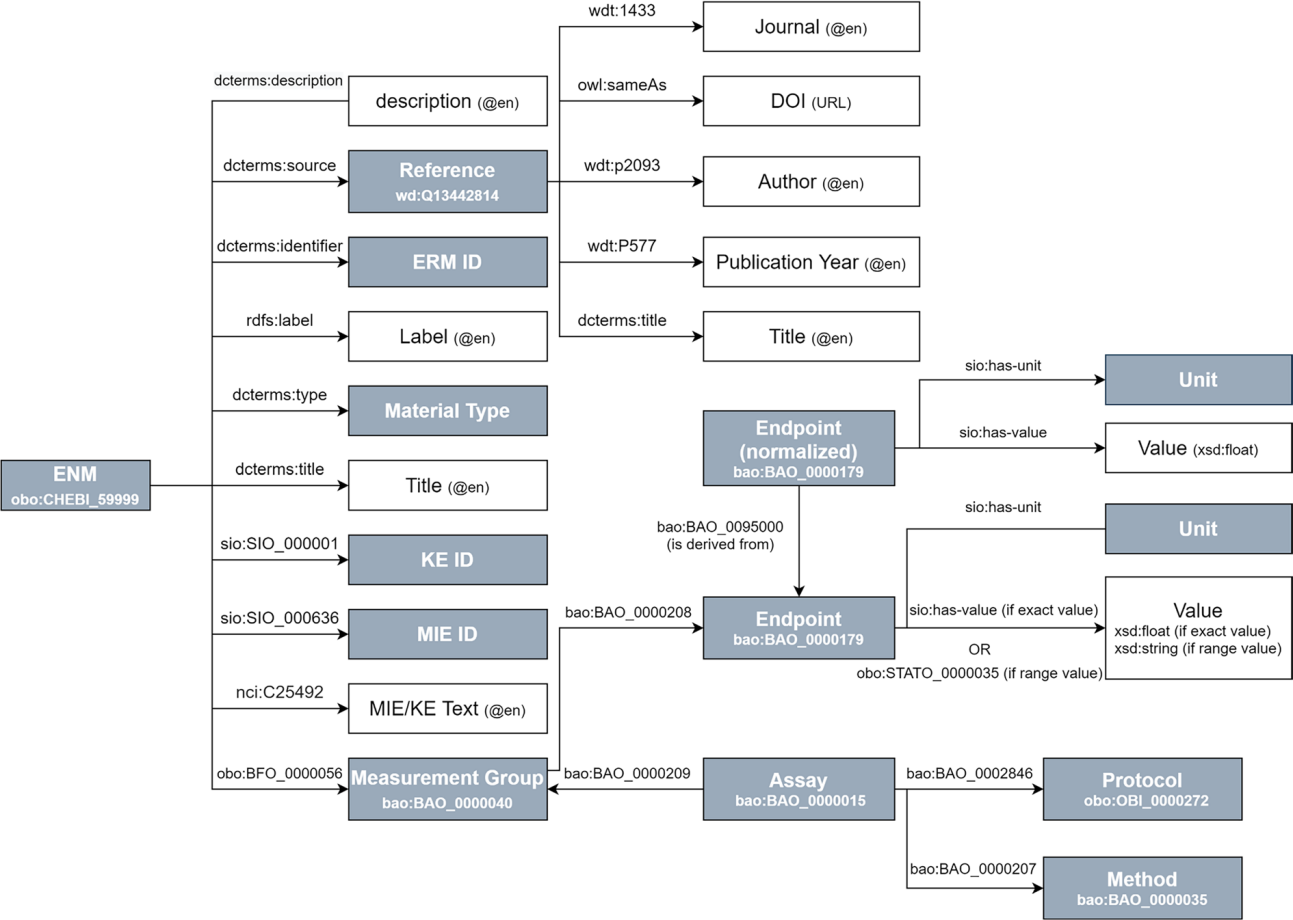
Graphical representation of the Resource Description Framework Schema for engineered nanomaterials (ENMs). Recognize subjects (labeled boxes) and predicates (labeled arrows). Objects can be literals (white boxes) or IRIs (blue boxes). The abbreviations of prefixes for the ontologies are defined in Table 1

Based on the spreadsheet Additional file 2 that resulted from the literature review, an RDF schema was developed and annotated using standard metadata vocabularies and specialized ontologies Fig. 1. Table 1 shows the ontologies used in developing the RDF along with their prefixes.

**Table 1:** Ontologies used in the NanoSafety RDF model

Ontology	Prefix	IRI
Ambit platform vocabulary	amb:	http://purl.enanomapper.net/
BioAssay ontology [37]	bao:	http://www.bioassayontology.org/bao#
Dublin core terms [38]	dcterms:	http://purl.org/dc/terms/
The experimental factor ontology [39]	efo:	http://www.ebi.ac.uk/efo/
eNanomapper ontology [32]	enm:	http://purl.enanomapper.org/onto/
National cancer institute ontology [40]	nci:	http://ncicb.nci.nih.gov/xml/owl/EVS/Thesaurus.owl#
Nanoparticle ontology [30]	npo:	http://purl.bioontology.org/ontology/npo#
Open biological and biomedical ontology [41]	obo:	http://purl.obolibrary.org/obo/
Web ontology language [42]	owl:	http://www.w3.org/2002/07/owl#
RDF schema [43]	rdfs:	http://www.w3.org/2000/01/rdf-schema#
Semanticscience integrated ontology [44]	sio:	http://semanticscience.org/resource/
Wikidata ontology [45]	wdt: wd:	http://www.wikidata.org/prop/direct/ http://www.wikidata.org/entity/

The transformation from the spreadsheet to RDF was performed using the RDF Mapping Language (RML). RML is a scalable, general mapping language designed to map heterogeneous data structures onto RDF models [34]. The process starts with creating a text document that contains rules on how each element in the spreadsheet can be mapped to a corresponding semantic expression. That is done by defining a subject map specifying the IRI structure for the subject and a set of predicate-object pairs to create the triples semantifying each cell value in the spreadsheet. The mapping document can be created using either RML rules directly or by using YARRRML as an intermediate representation which is then converted to RML using the YARRRML Parser [35]. YARRRML is a human-readable text-based format derived from YAML, allowing researchers to extend and maintain the mapping documents with less effort. It also requires less experience in semantic web formats to deal with, and thus it was chosen to express the mapping rules. Using both the spreadsheet and the mapping document, the RMLMapper [36] tool was used to produce a set of triples, which were then loaded into a triple store for exploring and querying.

### Deployment of the SPARQL webservice

In order to make the data publicly accessible, a SPARQL endpoint was loaded with the RDF and made available through nanosafety.rdf.bigcat-bioinformatics.org/ . The SPARQL endpoint is running on top of a Virtuoso triple-store deployed using Docker. The developed Docker image runs both Virtuoso and an Apache web server in one container, and it facilitates data import into Virtuoso using a custom-made bash script.

Furthermore, a SNORQL User Interface was developed to easily use SPARQL queries and share results (nanosafety.rdf.bigcat-bioinformatics.org/). This webpage includes an example query panel that is auto-populated with SPARQL queries stored in GitHub (github.com/h2020-riskgone/SPARQLQueries).

### Use cases of the RDF

To test the applicability of the NanoSafety RDF, a Jupyter Notebook was created. The data was queried using SPARQL queries to explore the contents, generate statistics, create graphical overviews, and highlight potential uses of the NanoSafety RDF. The Jupyter Notebook containing the SPARQL queries and code to make all figures are available on GitHub (https://github.com/h2020-riskgone/Nanomaterial-RDF/blob/main/nanoMIE.ipynb).

Various SPARQL queries were executed to explore parts of the data and utilize the interoperability of RDF by performing federated SPARQL queries with the AOP-Wiki, which contains more extensive information about the AOPs that are initiated by some of the ENMs. First, general information is queried about the presence of ENM types, their physicochemical characteristics, and the MIEs that were annotated for all ENMs. One of the MIEs, DNA damage (https://aopwiki.org/events/1194), was selected as a use-case for further exploration because the RDF contains sufficient information for this case, but DNA damage as MIE or KE is a rather specific effect and not a side product of various mechanisms of action. Using the RDF, the MIE of DNA damage was linked to material types and physicochemical characteristics, and potential AOs that can be caused by DNA damage were explored by including information contained in the AOP-Wiki RDF [33].

## Results

### The RDF schema and SPARQL endpoint

The Nanosafety RDF aims to follow the data models provided by the NPO [30] and the BioAssay Ontology (BAO) [37], two ontologies also reused in the eNM ontology [32]. Other ontologies used are the ChEBI ontology [29] and Vocabulary of Interlinked Datasets (VoID) [46]. Properties were added from the BAO, Chemical Information Ontology [47], NPO [30, 31], and Semanticscience Integration Ontology [44].

This activity resulted in a model where the following entities were modeled as typed resources (i.e., have an rdf:type triple):

- materials are typed as chemical substance (CHEBI: obo:CHEBI_59999)
- material types are typed with the ChEBI, NPO or eNM ontology
- assay readouts are typed as a measurement group (BAO: obo:BAO_0000040)
- individual endpoints are typed as an endpoint (BAO: bao:BAO_0000179)
- the procedure methods are typed as a protocol (obo:OBI_0000272)
- data sets are typed as data set (VoID: void:Dataset).

As can be seen in Fig. 1, each ENM is linked to a reference with dcterms:source. A reference contains the DOI of the article, article title, journal, publication year and author information. The ERM identifier is linked through dcterms:identifier and the semantic annotations of material type through dcterms:type. The dcterms:title corresponds to a label we provided, while rdfs:label is the name of the ENM used in the article. The MIEs mentioned in the paper were linked to MIE and KE identifiers of the AOP-Wiki (see Section "Literature review and dataset creation"). While sio:SIO_000001 (which stand for “is related to”) links the ENM to all KE identifiers from AOP-wiki, sio:SIO_000636 (which means “is trigger for”) only results in those KE ID’s that are labeled as MIE in AOP-wiki. The name used for the KE reported in the paper is linked with nci:C25492 (“effect”). The ENMs participate in (BFO_0000056) a measurement group (bao:BAO_0000040), linking to the measured endpoint (bao:BAO_0000179) and assay (bao:BAO_0000015). Starting from the measured endpoints (which are the physicochemical properties of ENMs such as primary size, hydrodynamic diameter, shape, zeta potential, etc.), their values and units can be explored with the predicates sio:has-value and sio:has-unit, respectively. For each assay, the protocol (obo:OBI_0000272) can be found with the bao:BAO_0002846 predicate. For six physicochemical properties, the data was normalized and added to the RDF using the predicate bao:BAO_0095000 (which means “is derived from”).

**Fig. 2:**
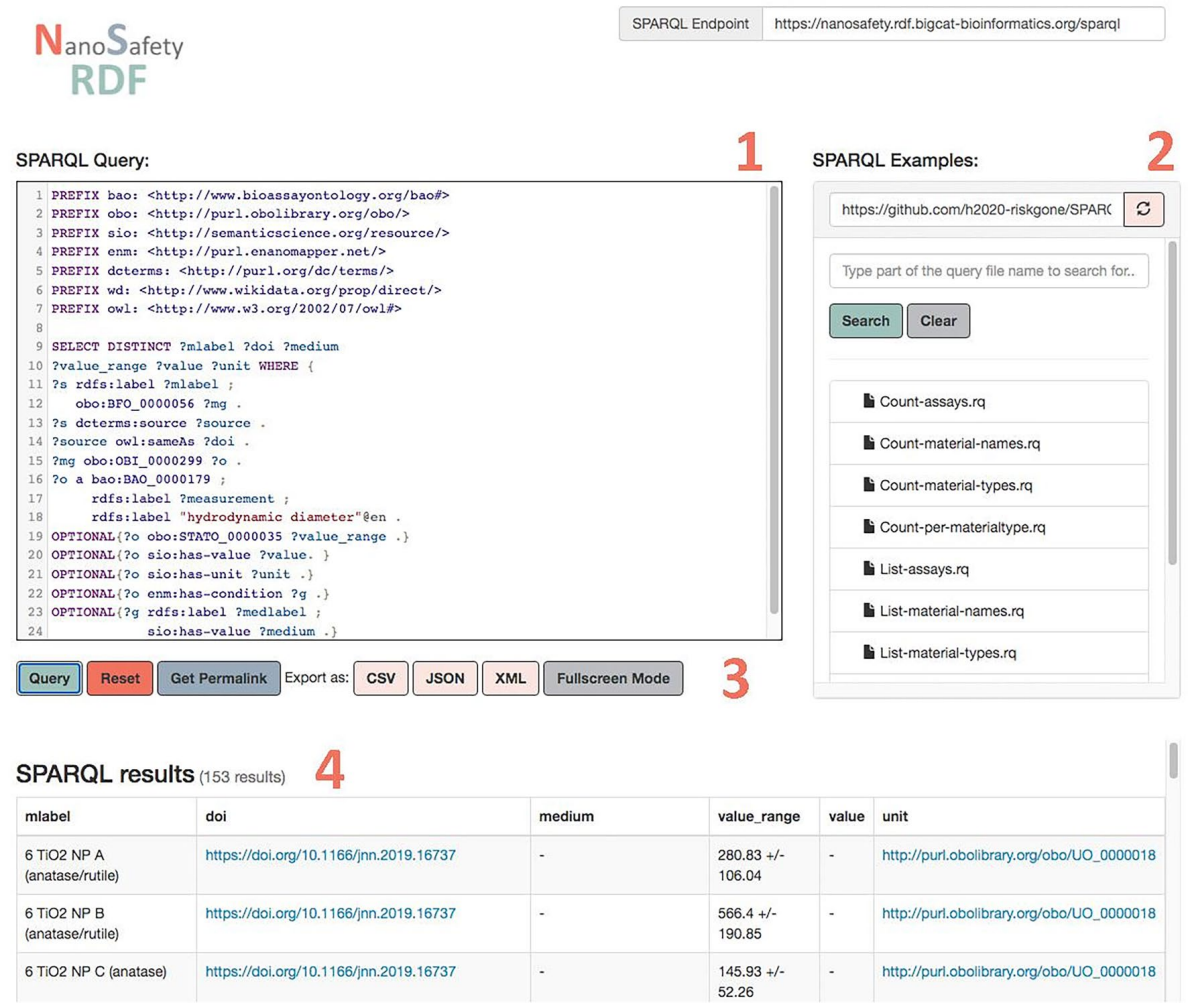
Screenshot of the SNORQL User Interface, consisting of a query box (1) and an example query panel (2) that is auto-populated with SPARQL queries stored in Github. The data can be queried through this interface as exports in various formats (.csv, .json, .xml, etc.) (3) or to return tables in a HTML page (4)

A screenshot of the SPARQL endpoint is shown in Fig. 2. The SNORQL User Interface consists of a query box and an example query panel that is auto-populated with SPARQL queries stored in GitHub. The data can be queried through this interface as exports in various formats (.csv, .json, .xml, etc.) or to return tables in an HTML page.

### Exploring the contents of the nanosafety RDF

**Fig. 3:**
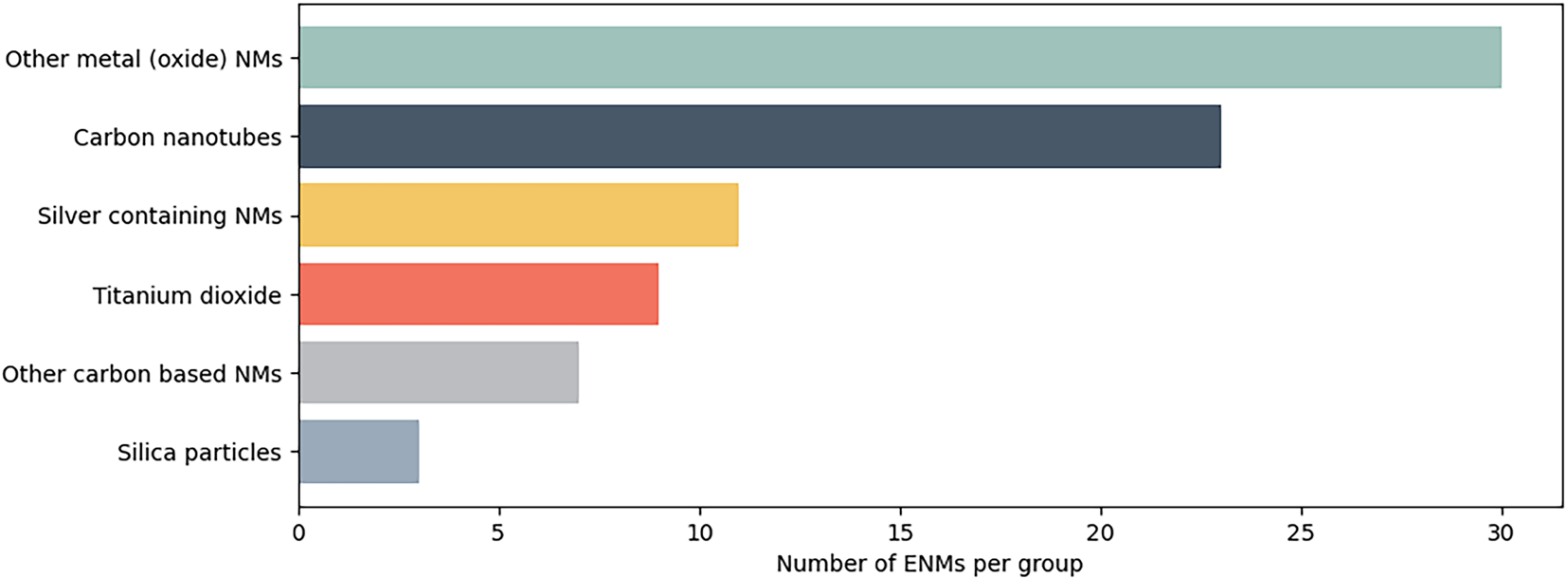
Distribution of the nanomaterials in the dataset, for details see Additional file 1: Table S1

Using the SPARQL endpoint Fig. 2, SPARQL queries were used to extract data from the RDF. In total, the generated RDF consists of 6,355,652 triples. Based on the 21 scientific papers, 87 unique ERMs were identified and included in the RDF, although 4 of these were micro or macro materials and were therefore excluded for the analyses in the rest of this paper. The ENMs were annotated according to their type using terms from the NPO, ChEBI, and eNM ontologies, resulting in a total of 35 types of ENMs. This number was obtained with the SPARQL query “Count-material-types.rq” from the example query panel. For the annotations, NPO was used 29% of the time, ChEBI 23%, and eNM 49%. The most reported ENMs in the selected literature include multi-walled carbon nanotubes, silver nanoparticles, and titanium dioxide nanoparticles see Fig. 3 that were reported by 6, 5 and 4 papers, respectively.

For Fig. 3, six groups are created based on the dcterms:title label using a Jupyter Notebook. These groups will be used throughout the following sections see Additional file 1: Table S1, but as can be seen in Section "Using ontology annotations for grouping of nanomaterials", the ENMs can also be grouped based on their ontology type. The group carbon nanotubes (CNTs) [23] consists of single-walled CNTs [9], multi-walled CNTs [8] and some specific (multi-walled) CNTs. The other carbon-based ENMs [7] contain graphene oxide [3] and graphene [2] among others. There are three types of silica particles [3]. The RDF contains information about nine titanium dioxide nanoparticles [9]. The silver-containing ENMs [11] contain standard materials like silver NM300K [2], generic materials like sphere-like silver nanoparticles [3] and specific particles like polyvinylpyrrolidone-coated silver nanoparticles [2]. Other metals and metal oxide ENMs [30] consist of about 10 rare earth metal ENMs, but also ENMs with iron oxide (3), cerium oxide (5), zinc oxide (5), copper (2), gold (1), cobalt (1), manganese oxide (1) and 27%- and 78%- zirconium oxide-doped cerium oxide nanoparticle (1–1).

**Fig. 4:**
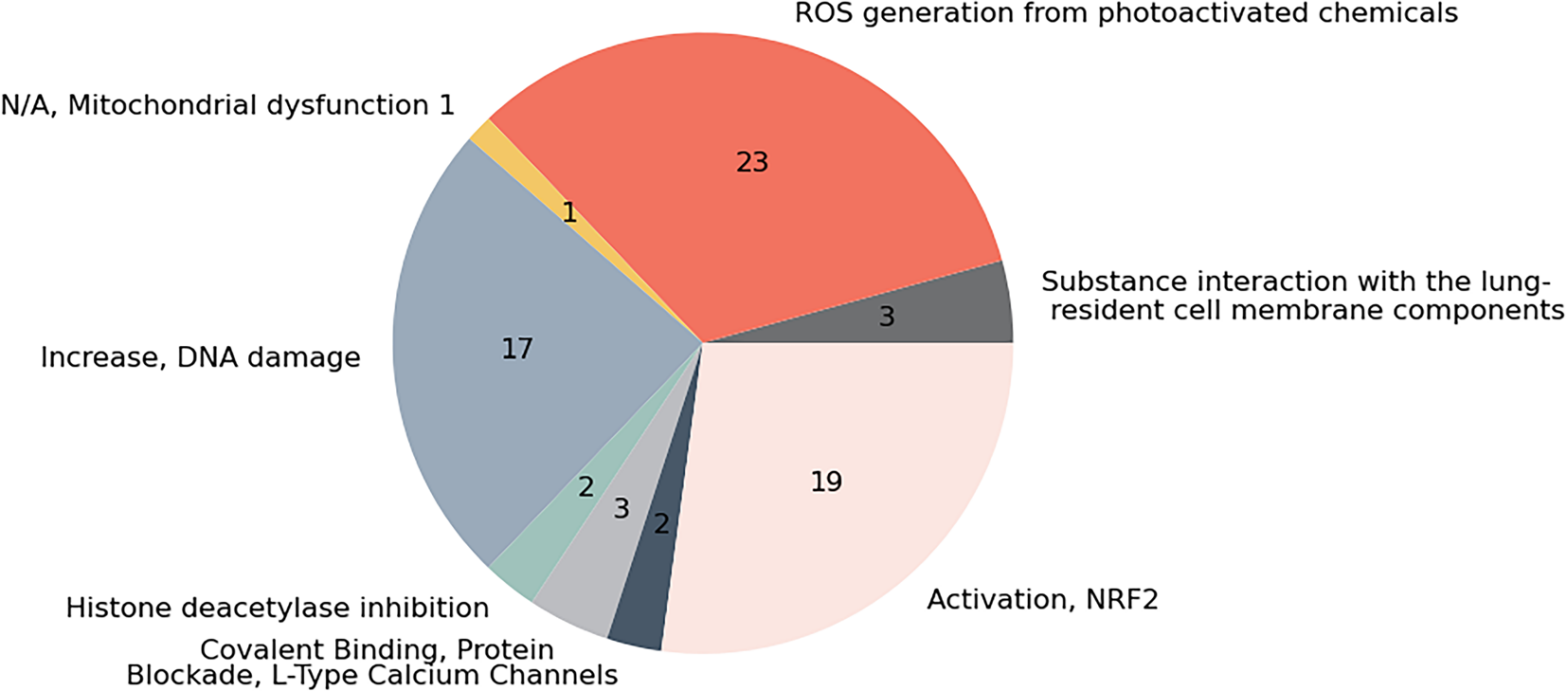
Number of unique ENMs (by counting unique ERM identifiers) coupled to each molecular initiating event (MIE). Each section indicates the proportion of ENMs (n = 53) that have been described to activate each individual MIE (n = 8)

Assessment of potential MIE activation showed that the majority of ENMs from the selected papers were described to cause either ROS production (33%), activation of Nrf2 (27%), and DNA damage (24%), as presented in Fig. 4. The AOP-Wiki describes 238 MIEs in total [48], of which 11 could be linked to the ENMs described in the literature, though 3 of these were induced by the same 23 ENMs (namely “ROS generation from photoactivated chemicals”, “Chronic reactive oxygen species” and “Increase, Reactive oxygen species production”) which were therefore only shown once in Fig. 4. In the papers, 43 molecular effects of the ENMs were mentioned, 31 of which could be linked to 64 KEs in AOP-Wiki of which 11 are MIEs see Table 2.

**Table 2:** Molecular effects of the ENMs mentioned in the analysed papers (first column), linked to 64 KEs in the AOP-Wiki (AOP-Wiki IDs of the KEs are reported in third column) of which 11 are MIEs (AOP-Wiki IDs of KEs that are MIEs are reported in the second column)

Molecular effects described in paper	AOP-Wiki ID—MIE	AOP-Wiki ID—KE
Activating the NLRP3 inflammasomes	NA	1895
Activation of Nrf2 signaling pathway and increased catalase activity	478	478
Activation of post-transcriptional mechanism	NA	NA
Activation of signaling molecules	NA	NA
Alter genome methylation	NA	1773;1778;1622;1827;1620
Alveolar Macrophage activation	NA	1198;1754
Apoptosis	NA	55;1262;1513;1365;1817;1825;1864
Apoptotic stimuli	NA	55;1262;1513;1365;1817;1825;1864
Carcinogenicity	NA	885;1556;1395;1670;1193;1839;1651
Cell cycle regulation/progression/control	NA	854;1555;870;269
Cellular sensing of substance or substance induced damage	1437	1437;1459
Cellular sensing of the substance-induced Damage resulting in the release of danger signals	1437	1437;1459
Condensed cellular size with reduced numbers of protrusions	NA	NA
Cytoskeleton remodeling	NA	NA
Decreased inflammatory response	NA	152
Direct stress of epithelium	NA	1586
Disrupt macrophage function	NA	NA
DNA damage	1194	1461;1551;1194;1766;1635;1669
Genotoxicity	1194	1461;1551;1194;1766;1635;1669
Histone modifications	1502	1502;1503
Increased the number and length of protrusions	NA	NA
Inducing membranolysis	NA	169;1498
Induction of IL1β and TNFα	NA	1492;134;1579;1577;1496
Induction of IL6	NA	87;1493
Induction of oxidative stress (CoO > Fe3O4 ≫ SiO2)	1753;1592;257	1364;257;1115;249;1278;1753;1592
Inhibition of cellular receptors (dopaminergic, adrenergic, cannabinoid)	NA	1083
Interact with skin proteins	396	396
Interaction with lung cells	1495	1495
Interference with signaling molecules	NA	NA
Interruption of calcium sensing receptors	1529	1529
Lysosomal injury	NA	898;831;1540
Mitochondrial dysfunction	177	177
No effect	NA	NA
Production of free radicals	1753;1592;257	1364;257;1115;249;1278;1753;1592
Proliferation	NA	854;1555;870;269
Promoted activities of SOD and Gpx	NA	NA
Pulmonary and cardiac effects	NA	NA
Pulmonary inflammation	NA	1438
Reactions with endogenous molecules	NA	NA
ROS (increases in the levels of malondialdehyde (MDA))	1753;1592;257	1364;257;1115;249;1278;1753;1592
ROS production	1753;1592;257	1364;257;1115;249;1278;1753;1592
ROS production through endoplasmic reticulum stress	1753;1592;257	1364;257;1115;249;1278;1753;1592
Surface silanol disorganization	NA	NA

During the literature review phase, sixteen different physicochemical properties were extracted, if reported in the paper. These properties were collected in the spreadsheet Additional file 2, and the most documented properties were converted to RDF, including the primary size, hydrodynamic diameter, shape, diameter, specific surface area, bundle diameter and zeta potential. Additional file 1: Table S2 shows the methods used to obtain each physicochemical property. In addition to the original data, normalized data were derived from the original data and added to the RDF.

Based on the reviewed literature, the most frequently reported physicochemical property was the shape, reported for 65 out of the 83 ENMs (78%), followed by the hydrodynamic diameter, primary size and zeta potential at 75%, 70% and 70%, respectively see Table 3. The shapes are distributed as follows: of the ENMs in the RDF 37 are spherical, twenty-one are tube-shaped, five are sheets and the RDF contains also one plate- and one cube-shaped ENM.

**Table 3:** Overview of physicochemical data with the number of ENMs (extracted by counting the unique ERM identifiers) that report a property and the min, max and mean value of these properties. When applicable, the medium is indicated

Physicochemical property	Medium	Nr. of ERM IDs	Min	Max	Average
Hydrodynamic diameter (nm)	All	62	9.0	3431.7	385.5
	Water	32	55.2	2007.8	646.3
Primary size (nm)		58	4.6	5730.0	229.7
Zeta potential (mV)	All	58	− 47.0	17.0	− 11.5
	Water	37	− 39.9	17.0	− 14.5
Diameter (nm)		22	0.8	1676.6	169.1
Surface area (m^2^/g)		16	6.9	316.5	102.4
Bundle diameter (nm)		8	5.0	13.0	8.4

### Application of the nanosafety RDF: Use case on DNA damage

Next, the application of the NanoSafety RDF was explored with a specific focus on one of the MIEs, DNA damage, which is a widely studied adverse effect of ENMs. By utilizing the NanoSafety RDF framework, we aim to get insights into the grouping of ENMs based on their ontological annotations, followed by exploring the effects that can be related to specified physicochemical properties, and finally extend our knowledge of relevant AOPs that involve DNA damage.

#### Using ontology annotations for grouping of nanomaterials

One of the advantages of using RDF in combination with ontology annotation is that the ontological tree structure can be used to group the materials using their annotation of material type. For example, MWCNTs and SWCNTs can be grouped by the parent term CNTs, or information related to all metal oxides can be queried at once. This is a useful feature that makes exploring groups of related nanomaterials possible. For example, to get all silver nanoparticles in the RDF, the following query takes the ontology term we assigned to each nanomaterial (dcterms:type ?t) and queries for the parent of this term (rdfs:subClassOf^*^ ?parent), filtering it to be a silver particle.

We can also return all literature sources in the RDF that describe any type of nanoparticle. For example for all CNTs, this amounts to nine papers for the 23 different CNTs in the dataset.

#### Analyzing physicochemical properties of nanomaterials

**Fig. 5:**
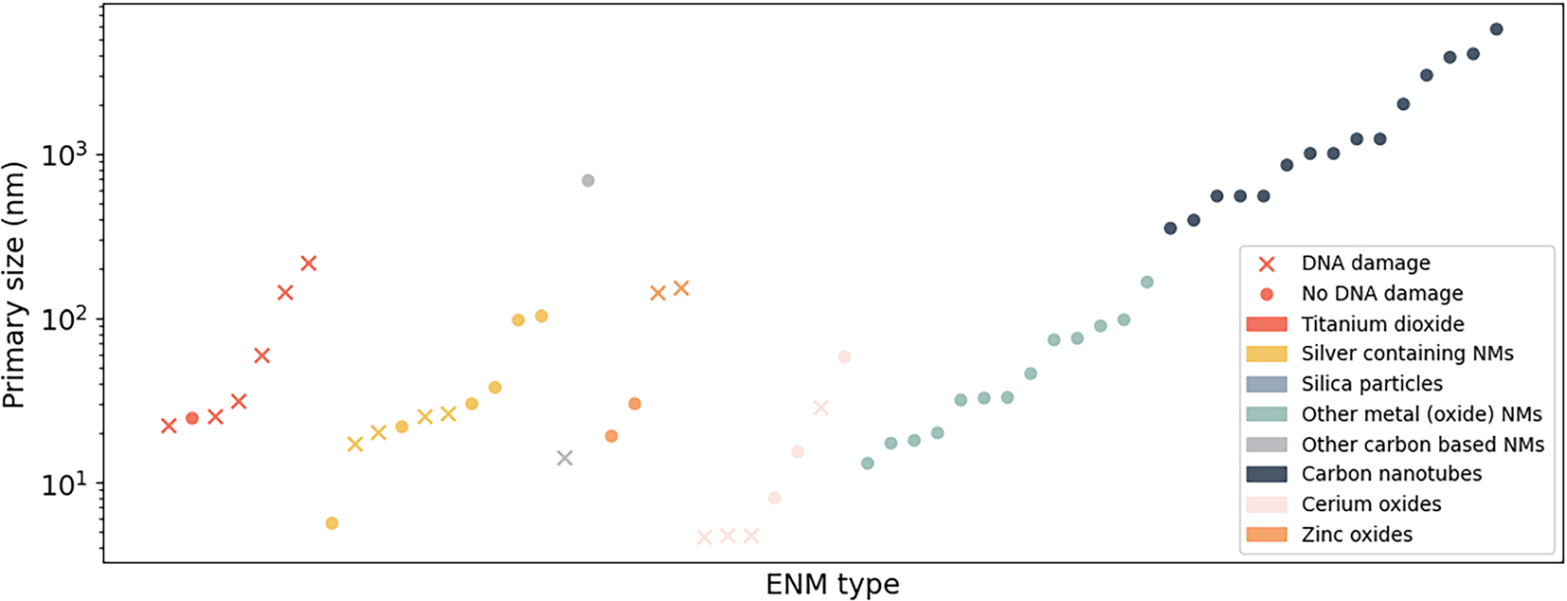
Overview of primary size values in RDF. Marker color based on material type with between brackets (i) the number of ENMs of this type that cause DNA damage, (ii) the number of this type with a primary size value: red titanium oxides (6/7), yellow silver ENMs (4/10), gray carbon ENMs (1/2), orange zinc oxides (2/4), pink cerium oxides (4/7), green metal oxides (0/13), blue CNTs (0/15), marker shape based on results of DNA damage positive (X) or negative (O)

As illustrated, SPARQL queries enable the extraction of information, such as the physicochemical properties of ENMs, facilitating the plotting of primary size values, as shown in Fig. 5. The shape of the point marker indicates the impact of each ENM on DNA damage, and the nanomaterial type is color coded similar to Fig. 3 (with the addition of zinc and cerium oxides colored orange and pink, respectively). The obtained data suggest that for, e.g*.*, silver nanoparticles (shown in yellow) the ENMs with a small primary size value are more likely to cause DNA damage (below 26 nm, 4/6 cause DNA damage, while 30 nm and above 0/4), while, titanium dioxide nanoparticles seem to cause DNA damage regardless of their size.

When looking at the fractions in the Fig. 5 caption in more detail, we see that, according to the data in our RDF, no CNTs cause DNA damage (0/15), while for titanium dioxide particles 6 out of the 7 titanium dioxide particles, for which a primary size value was reported in literature, do cause DNA damage. Similarly, a large fraction of the cerium oxides, zinc oxides and silver-containing nanomaterials in our dataset cause DNA damage, while our RDF does not contain data on DNA damage for, e.g., iron oxides or other metal oxides (0/13).

#### Exploring the biological effects of nanomaterials and potential adverse outcomes

The SPARQL endpoint can also be used to explore the potential AOs of ENMs as a result of the MIEs described in literature, by extending the information with content from the AOP-Wiki. This was done in the query “AOs-from-MIEs.rq” from the example query panel, which explores the downstream effects of the proposed MIEs in more detail.

**Fig. 6:**
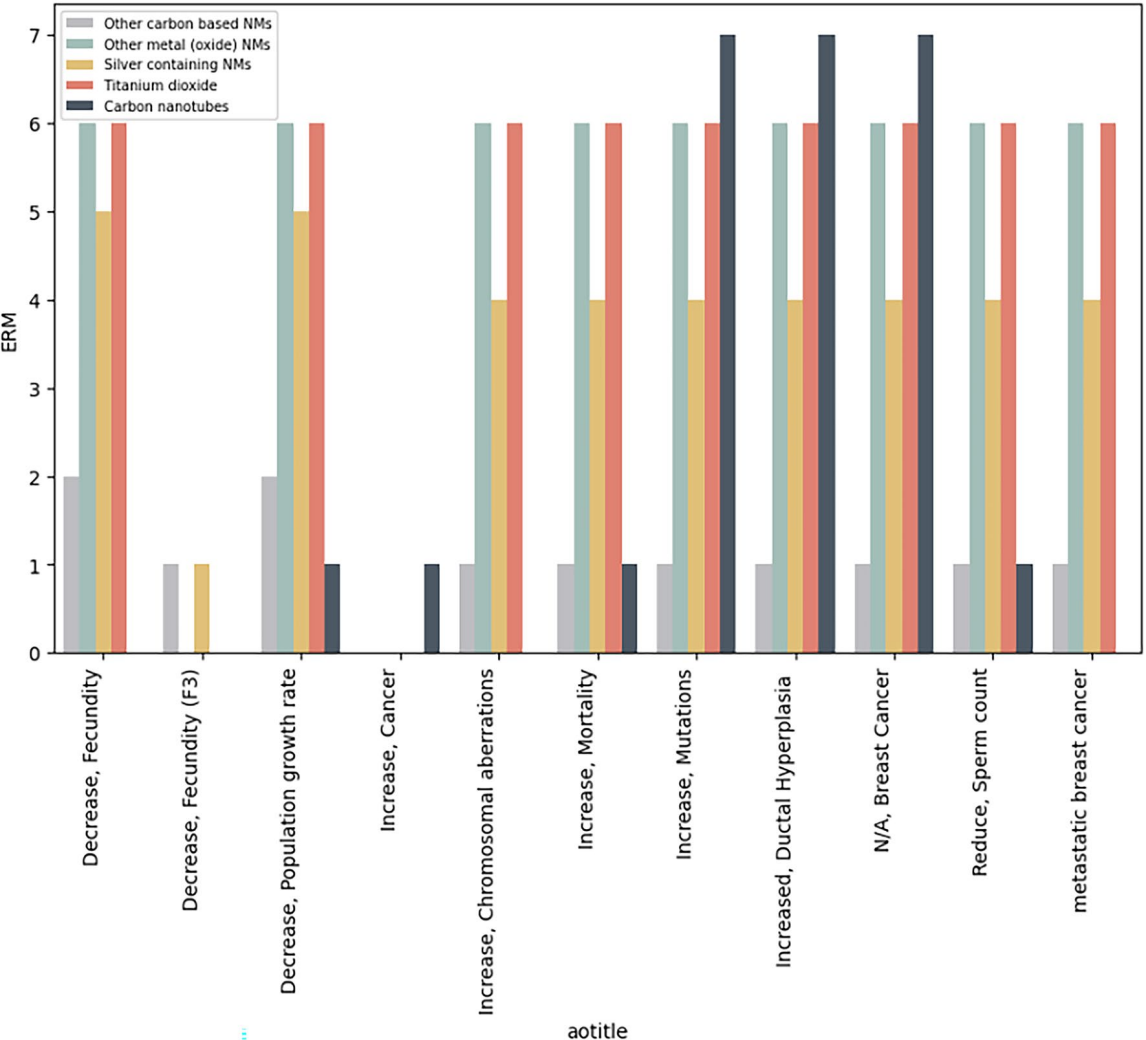
Number of ENMs in the RDF that causes a DNA-related MIE per resulting AO (on x-axis), split on type (CNTs (total number from this query n = 7), other carbon-based ENMs (n = 2), metal (oxide) ENMs (n = 6), Silver containing ENMs (n = 5), titanium dioxide (n = 6)

The query returns 280 results for 26 unique ENMs, with 13 MIE-AO combinations Table 4. Figure 6 shows the association of each specific AO with its corresponding ENM, irrespective of the MIEs outlined in Table 4. Grouping the ENMs, as described in Section. "Exploring the contents of the NanoSafety RDF" and Additional file 1: Table S1, resulted in seven CNTs, two other carbon-based ENMs, five silver-containing ENMs, six titanium dioxide nanoparticles and six other metal (oxide) ENMs.

**Table 4:** Overview of DNA-related MIEs and the AOs they cause. ERM counts correspond to the number of ENMs in the RDF that cause the MIE-AO combination

MIE title	AO title	ERM count
Increase, DNA damage	Increase, Mutations	24
	Increased, Ductal Hyperplasia	24
	N/A, Breast Cancer	24
	Decrease, Population growth rate	18
Increase, DNA methyltransferase inhibition	Decrease, Population trajectory	19
	Decrease, Fecundity	19
	Decrease, Fecundity (F3)	2
Alkylation, DNA	Reduce, Sperm count	18
	Increase, Cancer	1
Increase, formation of DNA photoproducts	Increase, Mortality	18
Increased, DNA damage and mutation	Metastatic breast cancer	17
Increase, oxidative damage to DNA	Increase, Chromosomal aberrations	17
	Increase, Mutations	17

As an example, Table 4 highlights that 18 ENMs could cause an AOP (https://aopwiki.org/aops/444) that starts with an “increase in DNA damage” (MIE) which ultimately leads to a “decrease in population growth rate” (AO). Figure 6 indicates that this effect could be caused by all metal (oxide), silver-containing and titanium dioxide ENMs in this dataset, while only one out of the seven multi-walled carbon nanotubes in the dataset might lead to this specific AO.

## Discussion

From the experimental data presented in literature, it is often difficult to fully assimilate the interaction between ENMs and biological structures or systems, which limits insights into the MIEs/KEs they can trigger and inhibits the development of nano-related AOPs. Here, we present a proof of principle of implementing semantic web approaches for reporting ENMs, their physicochemical characteristics, and their interactions with biomolecules and biological systems. It expands on the work initiated by Murugadoss et al. (2021) where a scientific literature analysis was performed to connect ENMs with existing AOPs [27]. That resulted in the identification of ENM-relevant MIEs and KEs, which were used to explore and repurpose chemical-based AOPs and KEs for ENMs to set up in vitro testing strategies, which was demonstrated with two case studies.

Although the approach used here to create the dataset and the RDF-generated form has made the information more FAIR, it has limitations to consider. First, the number of papers analyzed is limited when compared to the increasing number of scientific papers on nanotoxicology that are published every year, which was close to 6000 publications in 2020 [49]. As a proof of principle 21 papers were investigated which restricts the scope of the findings. To obtain a more comprehensive understanding of the relationship between ENMs and their properties with specific AOPs, additional papers could be included for data extraction. However, this goes beyond the scope outlined in this proof-of-principle paper. Second, data quality poses a significant challenge in the curation process. The adequacy, completeness, accuracy, reliability, relevance, and variability of the reported data in the literature all contribute to its overall quality. It is crucial to evaluate these parameters before proceeding with data analysis [50]. Different scoring or weighing systems, such as the ToxRTool [51], the GUIDEnano tool [52] or CRED [53] evaluation methods, can be utilized for qualitative or quantitative data quality assessments. The third limitation relates to the quality of contents in the AOP-Wiki, where the same key events often have different identifiers. AOPs and their KEs provide simplified descriptions of biological processes, but inconsistencies can arise due to overlapping or conflicting definitions across different ontologies. Therefore, careful consideration is required when utilizing AOP-Wiki for data analysis. The limitations that result from inconsistencies and duplications of AOPs pose a limitation to their utility, underscoring the need for ongoing efforts to enhance their quality and comprehensiveness to further refine and strengthen future investigations in the field. Additionally, exploring potential linkages with other databases, such as molecular pathway databases, could offer valuable and broader insights. Fourth, limitations regarding data accessibility present a challenge. Not all data dealing with ENMs toxicity testing, especially "no effect" data, are published or easily accessible. Even publishing the chemicals discussed in an article in a standardized manner is rare [54, 55]. Additionally, industry-held data are hardly accessible due to confidentiality. Although efforts are being made to promote open access and adherence to the FAIR guiding principles [20], depending on the type of data, repositories are not yet fully established and, when available, are not always completed. Finally, the labor-intensive nature of data curation from scientific literature presents difficulties in scaling up the process and covering a large volume of relevant papers. This challenge is not unique to extracting information from journal articles and is also observed in other initiatives focusing on scientific reporting of nanomaterials and characteristics [56]. To overcome this limitation, the scientific community has explored automated methods, such as machine learning and text mining to extract information from unstructured text [57–59]. These techniques aim to improve efficiency and reduce inconsistencies in data extraction. However, manual curation can also introduce discrepancies in reporting and interpretation due to the involvement of multiple individuals with different interpretations and terminologies. Efforts have been made to mitigate these inconsistencies by addressing synonyms and rephrasings. Overall, the automatic extraction of information about papers remains a task that requires further development and improvement.

The NanoSafety RDF offers various applications and potential for future development. It demonstrates its utility through a use-case focused on the physicochemical properties of nanomaterials and their potential to cause DNA damage. The use of SPARQL queries on the developed RDF allows for the exploration of the relationships between nanomaterial properties and biological effects. However, the size of the dataset limits the avail of the conclusions that can be drawn from it. For example in the analysis of the effect of primary size on the ability to activate particular types of MIEs, key information (like dissolution rate, agglomeration) which is not included in the dataset are also required to refine the analysis [60]. The availability of a SPARQL endpoint facilitates the usability of the data, allowing users to interact with it through user-friendly web interfaces or computational means. The presented RDF schema can serve as a starting point for reporting nanomaterials and their properties. Another valuable application is the ability to provide feedback to the AOP-Wiki. By linking KEs and MIEs, gaps in existing AOPs can be identified, guiding additional data and research. Moreover, the identification of KEs associated with nanomaterials, but not explicitly described as MIEs in AOP-Wiki, presents an opportunity to propose new AOPs, highlighting areas where the understanding of nanomaterial adverse effects needs improvement. The NanoSafety RDF also allows for similarity searching, where researchers can explore patterns and correlations between physicochemical and ontological characteristics of nanomaterials. This capability could overcome major challenges in nanomaterial similarity assessment and therefore aids in further research and analysis in the field [61, 62].

In future directions, process automation is a key area of focus. The current manual approach to extracting nanomaterial information from scientific papers hinders scalability. However, advancements in text-mining approaches and APIs can automate or assist in the extraction process, significantly improving efficiency and enabling the extraction of a larger volume of data. Maintenance of the NanoSafety RDF is vital as the literature on nanomaterial characterization and potentially harmful effects continues to expand. Employing more comprehensive literature search and selection methods can ensure the inclusion of a broader range of nanomaterials and their characteristics in the RDF. Expanding the RDF content is another important aspect. Currently, the RDF schema includes only six properties due to the restricted reporting of physicochemical characteristics in scientific papers. To capture a more exhaustive understanding of nanomaterials, it is necessary to expand the range of properties included in the schema, facilitating the exploration of correlations between physicochemical properties and molecular effects.

One of the main take-home messages resulting from the described approach is the need for mutual agreement on minimal reporting requirements of physicochemical characteristics of ENMs and a standardized format for these data in future studies. Although it is well known that the physicochemical characteristics of ENMs determine their toxicokinetics and toxicodynamics [63–66], many studies on AO of ENMs provide not even basic data on ENMs size, shape, surface charge, surface functionalization or core composition. It is also crucial to consider the changes of ENMs properties such as aggregation, agglomeration, dissolution [67, 68] in different exposure conditions for the successful implementation of computational tools and models in risk assessment. Data reporting using templates and the use of identifiers for ENMs [28] are recommended to improve data consistency. Additionally, comprehensive characterization of ENMs’ physicochemical properties is essential for accurate risk assessment in nanotechnological applications [56].

## Conclusions

This work presented a proof of principle of implementing semantic web approaches for reporting ENMs and their interactions with biological events and resulted in an open dataset and a publically available SPARQL endpoint for exploration and query answering. The work has shed light on various aspects of nanosafety research, specifically in the context of extracting and analyzing information from the scientific literature. We explored the challenges posed by manual work in data curation and the limitations it imposes on scalability and consistency. Furthermore, we discussed the application of the NanoSafety RDF and its utility in investigating the physicochemical properties of nanomaterials and their potential effects. The RDF's accessibility through SPARQL endpoints and user-friendly interfaces offers researchers the opportunity to explore relationships between properties and biological effects, contributing to the advancement of nanosafety research in a safe(r) and sustainable manner. Looking ahead, we identified future directions and recommendations and emphasized the importance of standardized data reporting and the inclusion of key identifiers in nanosafety studies. Overall, this work has provided insights into the challenges, opportunities, and recommendations for advancing nanosafety research, showcasing the potential of semantic web approaches and computational tools in supporting the understanding of nanomaterials and their effects on human and environmental health.

### Supplementary Information

**Additional file 1: Figure S1. **Example of an ERM identifier as added to the registry Turtle. **Table S1. **Overview of nanoparticles (ENMs) in the NanoSafety RDF and how they are divided into the six groups shown in Figure 3 and 6. Names from the papers are abbreviated using CNTs for carbon nanotubes and NP for nanoparticles. **Table S2. **Overview of the methods and instruments used to obtain each physicochemical property.

**Additional file 2:** Spreadsheet with raw data. The main sheet with the final data is the seventh sheet.

## Data Availability

The data on ENM characteristics and their toxicological effects come from peer-reviewed literature, of which metadata is stored in the created RDF and in the collection spreadsheet. The collection spreadsheet, containing all data that was converted into RDF is available as Additional file 2. The RDF has been archived in Zenodo (10.5281/zenodo.8076364). The annotations with MIEs and KEs were done with AOP-Wiki (https://aopwiki.org/). The federated SPARQL queries with the AOP-Wiki RDF utilized the AOP-Wiki SNORQL endpoint (https://aopwiki.rdf.bigcat-bioinformatics.org/sparql). The Jupyter notebook that produced the majority of the figures and tables has been made available on GitHub (https://github.com/h2020-riskgone/Nanomaterial-RDF). The documentation on the deployment and loading of the RDF in a SPARQL endpoint is available in GitHub (https://github.com/h2020-riskgone/nanosafety-virtuoso-httpd-docker). The code used to create the RDF including the RML mapping and the output RDF is available on GitHub (https://github.com/h2020-riskgone/Nanomaterials-MIE-interactions-RDF) and a release is archived on Zenodo with DOI (10.5281/zenodo.8075705).

## Abbreviations

AO: Adverse Outcome
AOP: Adverse outcome pathway
CNT: Carbon Nanotube
ENM: Engineered nanomaterial
IATA: Integrated Approach to Testing and Assessment
KE: Key Event
MIE: Molecular initiating event
NAM: New Approach Methodology
PhysChem: Physicochemical
RA: Risk assessment
RDF: Resource description framework
RML: RDF mapping language
SPARQL: SPARQL protocol and RDF query language
SWNT: Single-Walled Carbon Nanotube
