# Supplementary material for: From papers to RDF-based integration of physicochemical data and adverse outcome pathways for nanomaterials
Source: J Cheminform. 2024 May 1;16:49. doi: 10.1186/s13321-024-00833-0 (PMC11064368; doi:10.1186/s13321-024-00833-0)
Supplement: Supplementary file 1 — Additional file 1: Figure S1. Example of an ERM identifier as added to the registry Turtle. Table S1. Overview of nanoparticles (ENMs) in the NanoSafety RDF and how they are divided into the six groups shown in Figure 3 and 6. Names from the papers are abbreviated using CNTs for carbon nanotubes and NP for nanoparticles. Table S2. Overview of the methods and instruments used to obtain each physicochemical property. [file 13321_2024_833_MOESM1_ESM.docx]

**Figure S1:** Example of an ERM identifier as added to the registry Turtle.

erm:ERM00000099 a obo:CHEBI_59999 ;

rdfs:label "RiskGONE Literature NM 1" ;

dct:creator wd:Q61965386 ;

foaf:page

<https://h2020-riskgone.github.io/riskgone-materials/RiskGONE_Literature_NM.html> .

**Table S1:** Overview of nanoparticles (ENMs) in the NanoSafety RDF and how they are divided into the six groups shown in Figure 3 and 6. Names from the papers are abbreviated using CNTs for carbon nanotubes and NP for nanoparticles.

| **carbon nanotubes (23)** |  | **silica particles (3)** |  | **other metal oxides II (22/31)** |  |
| --- | --- | --- | --- | --- | --- |
| single-walled CNTs | 9 | crystalline silica particles | 1 | dysprosium oxide NP | 1 |
| multi-walled CNTs | 8 | silica oxide NP | 1 | erbium oxide NP | 1 |
| multi-walled CNTs aerosol | 1 | silica quartz particles DQ12 | 1 | europium oxide NP | 1 |
| multi-walled CNTs aminated | 1 |  |  | Fe2O3 NP | 1 |
| multi-walled CNTs carboxylated | 1 | **silver containing ENMs (11)** |  | Fe3O4 NP | 2 |
| multi-walled CNTs polyethyleneimine | 1 | polyvinylpyrrolidone-coated silver NP | 2 | gadolinium oxide NP | 2 |
| multi-walled CNTs pegylated | 1 | silver NM300K | 2 | gold NP | 1 |
| carbon nanotubes | 1 | silver cube NP | 1 | lanthanum oxide NP | 1 |
|  |  | silver NP | 2 | manganese oxide NP | 1 |
| **other carbon based ENMs (7)** |  | silver plate NP | 1 | neodymium oxide NP | 1 |
| graphene oxide | 3 | spherelike silver NP | 3 | samarium oxide NP | 1 |
| graphene | 2 |  |  | ytterbium oxide NP | 1 |
| fullerene-C60 | 1 | **other metal oxides I (9/31)** |  | yttrium oxide NP | 1 |
| printex 90 carbon black NP | 1 | cerium oxide NM212 | 1 | zinc oxide NP | 3 |
|  |  | cerium oxide NP | 4 | zinc oxide NP NM110 | 1 |
| **titanium dioxide (9)** |  | 27% zirconium oxide-doped cerium oxide NP | 1 | zinc oxide NP NM111 | 1 |
| titanium dioxide NP | 9 | 78% zirconium oxide-doped cerium oxide NP | 1 | cobalt oxide NP | 1 |
|  |  |  |  | copper NP | 2 |

**Table S2:** Overview of the methods and instruments used to obtain each physicochemical property.

| **Physicochemical property** | **Method** | **Instrument** | **Number of ENM** |
| --- | --- | --- | --- |
| primary particle size | TEM | Hitachi H-7500 | 46 |
|  | TEM/EDS | Philips CM20 | 1 |
|  | TEM/AFM | JEOL 100CX | 8 |
|  | Method not specified |  | 3 |
| hydrodynamic diameter  (medium water) | DLS | HT-DLS, Dynapro Plate Reader, Wyatt Technology | 36 |
|  | Photomicrograph | CytoViva | 3 |
|  | Method not specified |  | 24 |
| bundle diameter | TEM |  | 8 |
| diameter | TEM |  | 8 |
|  | TEM/EDS | Philips CM20 | 1 |
|  | TEM/AFM | JEOL 100CX | 4 |
|  | UV-Vis |  | 8 |
|  | Method not specified |  | 1 |
| shape | TEM |  | 40 |
|  | TEM, SEM |  | 1 |
|  | Method not specified |  | 44 |
| zeta potential |  | Malvern ZetaSizer Nano-ZS | 39 |
|  | ELS |  | 7 |
|  | Method not specified |  | 12 |
| specific surface area | BET |  | 14 |
|  | Method not specified |  | 2 |
